# The Relationship Between PD-1(rs2227981) and PD-L1(rs2890658) Polymorphisms and Urothelial Cell Carcinoma

**DOI:** 10.7759/cureus.48120

**Published:** 2023-11-01

**Authors:** Sa Tin Myo Hlaing, Putri Damayanti, Khine Zin Aung, Hiromasa Tsukino, Takuji Hinoura, Yoshiki Kuroda

**Affiliations:** 1 Department of Public Health, Faculty of Medicine, University of Miyazaki, Miyazaki, JPN; 2 Department of Urology, Junwakai Memorial Hospital, Miyazaki, JPN

**Keywords:** pd-l1, pd-1, japanese population, polymorphism, urothelial cell carcinoma

## Abstract

Background

Urothelial cell carcinoma, which is believed to develop from the urothelium (transitional epithelium), is the most common aggressive tumor and accounts for the ten most prevalent cancers in the world. The risk factors for urothelial cell carcinoma are aging, smoking, gender, and genetic alternations. Programmed cell death1 (PD-1) has been widely described as a negative regulator of T-cells by sending inhibitory signals to the T-cell. Through PD-1 binding with PD-L1 (ligand for PD-1), an inhibitory signal is propagated to the T cell. The polymorphisms of PD-1 and PD-L1 lead to an efficient T-cell response and affect an anti-tumor reaction. The polymorphisms of PD-1 and PD-L1 could also affect the carcinogenesis of human cancer, including urothelial cell carcinoma. Therefore, in this study, we evaluated the relation between PD-1(rs2227981) and PD-L1(rs2890658) polymorphisms and the carcinogenesis of urothelial cell carcinoma.

Materials and methods

This study was conducted using 211 healthy controls and 256 cases of urothelial cell carcinoma among the Japanese population. The DNA samples were extracted from the peripheral white blood cells of each subject. The genotype was detected by using the Polymerase Chain Reaction-Restriction Fragment Length Polymorphism (PCR-RFLP) method.

Results

C/T (Adjusted OR 1.55, 95% CI:1.02-2.35) and C/T+T/T (OR 1.46, 95% CI:1.01-2.12) genotypes of PD-1 rs2227981 were significant and risk factors for urothelial cancer. Male with A/A genotype in PD-L1 and CT genotype in PD-1 has a significant higher risk factor compared with other genotypes (Adjusted OR 1.83, 95% CI:1.05-3.21).

Conclusions and discussion

We found that C/T(PD-1) and “A/A (PD-L1) and C/T(PD-1)” were predominant in urothelial cell carcinoma cases. This indicates that C/T(PD-1) and “A/A (PD-L1) and C/T(PD-1)” genotypes could increase susceptibility to urothelial cell carcinoma. However, since our findings indicated that the effects of PD-1 and PD-L1 polymorphisms included discrepancies, additional research will be needed to evaluate the relationship between human cancer and PD-1 and PD-L1 polymorphisms. This is the first study that seeks to find the relation between PD-1(rs2227981) and PD-L1(rs2890658) polymorphisms concerning urothelial cell carcinoma among the Japanese population.

## Introduction

Urothelial cell carcinoma, which is believed to develop from the urothelium (transitional epithelium), is the most common aggressive tumor and accounts for the 10 most prevalent cancers in the world [1]. The pelvis, ureter, bladder, and urethra are covered by the urothelium. Among urothelial cancers, bladder cancer could account for the majority [2]. Risk factors for urothelial cell carcinoma include aging, smoking, gender differences, and genetic alterations [3,4]. Polymorphism is the genetic alternations that attenuate two or more forms of a specific DNA sequence. Genetic polymorphism is the changes in genetic information between the same group. Genetic polymorphism exists in 1% or more of a population. Many such changes in base sequences are thought to have no effect on the body, but some may affect a person's appearance, such as eye color or hair color, or susceptibility to cancer. Moreover, some genetic alternations and single nucleotide polymorphisms (SNPs) can also influence the carcinogenesis of urothelial cell carcinoma [5-10].

Programmed cell death1 (PD-1, also known as CD279 or PDCD1), a member of the CD28-B7 co-stimulatory molecule superfamily, has been widely described as a negative regulator of T-cells by sending inhibitory signals to the T-cell. PD-1 protein is found on the surface of activated CD4-positive and CD8-positive T-cells, natural killer T-cells (NKT), B cells, activated monocytes, and various DC subsets [11].

The human PD-1 gene which is located on chromosome 2q27.3, encodes a type 1 trans-membranous glycoprotein protein of 50-55kDa [12]. The PD-1 protein is made up of two parts, one is an external immunoglobulin V domain and an intracellular domain with an immune receptor tyrosine-based inhibitory motif (ITIM), and the other is an immune receptor tyrosine-based switch motif (ITSM) [13]. When PD-1 binds with PD-L1 or PD-L2 (ligand for PD-1), the ITIM of PD-1 can be activated, and an inhibitory signal is propagated to the T-cell. That signal can attenuate T lymphocyte activation induce proliferation, and decrease cytokine release, leading to T-cell death [14-17]. Therefore, PD-1 protein plays an important function in suppressing inefficient or harmful immune responses and maintaining immunological tolerance. On the other hand, PD-1 is related to carcinogenesis, as it reduces the protective immune response [18]. Recently, a PD-1 monoclonal antibody has been invented for use as an anticancer agent [19].

The programmed cell death ligand 1 (PD-L1) gene that is located at the 9p24 chromosome is encoded by seven exons. The PD-L1 protein is made up of 290 amino acids and is one of the transmembrane type 1 glycoproteins that has IgV-like and IgC-like extracellular domains, a hydrophobic transmembrane domain, and a 30 amino acid cytoplasmic tail with unknown signal transduction capabilities. The PD-L1 protein is associated with cancer immune evasion [20-22]. Some studies have indicated that the abnormal expression of PD-L1 on the cell lines is related to the carcinogenesis of several tumors, such as cervical cancer, gastric cancer, and breast cancer [23-25].

Since the inactivation of PD-1 and PD-L1 genes has been associated with a critical role in the etiology of many cancerous conditions [26], the blockade of PD-1/PD-L1 interactions leads to an efficient anti-tumor T-cell response. However, the influence of PD-1 and PD-L1 polymorphism upon carcinogenesis has been unclear. Therefore, in this study, we evaluated the relation between one pair of co-inhibitory signaling molecule genes (PD-1(rs2227981) and PD-L1(rs2890658) genes) polymorphisms and the carcinogenesis of urothelial cell carcinoma.

## Materials and methods

This study was conducted using 211 healthy controls and 256 cases (patients with urothelial cell carcinoma) among the Japanese population. Cases were recruited from patients who were diagnosed with urothelial cell carcinoma histologically at the University of Occupational and Environmental Health Hospital and the University of Miyazaki Hospital. Controls were also recruited from patients without cancer at the University of Occupational and Environmental Health Hospital. We checked that cases and controls did not have any other malignancy or exposure to carcinogenic substances, toxic heavy metals, or radiation. We collected the physical conditions, history of illness, occupation, and smoking status of both controls and cases. The conditions of the research procedure were explained to all participants, and all participants gave written informed consent. This study was approved by the Ethics Committee of the Faculty of Medicine, University of Miyazaki.

Genotyping

The DNA samples were extracted from the peripheral blood of each subject. The DNA extractor WB Kit (Wako Pure Chemical Industries, Osaka, Japan) was used for the DNA extraction. The DNA extractor WB Kit was used frequently with other studies and is very safe because this kit does not use hazardous chemicals such as phenol and chloroform.

The Polymerase Chain Reaction-Restriction Fragment Length Polymorphism (PCR-RFLP) method was used for detecting genotypes of the PD-1(rs2227981) and PD-L1(rs2890658) genes. The primers of PD-1(rs2227981) were 5’-GTGTTCTCTGTGGACTATGG-3’(forward), 5’-CTGAGGAAATGCGCTGAC-3’(reverse) and the primers of PD-L1(rs2890658) were 5’-AATGGCTTGTTGTCCAGAGATG-3’(forward) and 5’-GTACCACA TGGAGTGGCTGC-3’(reverse). The PCR used in the first step was conducted using a KAPATaq Extra PCR kit (NIPPON Genetics Co., Ltd., Tokyo, Japan) under the following conditions.

The PCR conditions used for the PD-1(rs2227981) initially involved denaturation at 95 °C for 10 min, followed by 30 cycles of denaturation at 95 °C for 45 s, annealing at 60 °C for 40 s, and extension at 72 °C for 40 s, with the final extension at 72 °C for 10 min. The PCR for the PD-L1(rs2890658) gene was the initial denaturation at 95 °C for 4 min, followed by 35 cycles of denaturation at 95 °C for 40 s, annealing at 63 °C for 40 s, and extension at 72 °C for 40 s, with the final extension at 72 °C for 10 min. Both PCR products were digested by the restriction enzyme AluI for PD-1(rs2227981) and Ban II for PD-L1(rs2890658) (New England Biolabs Inc., Ipswich, MA, USA). The genotypes were detected by electrophoresis using 3% agarose gel. The lengths of the PCR products were 284bp and 553bp for PD-1(rs2227981) and PD-L1(rs2890658), respectively. The T allele of PD-1(rs2227981) is characterized by a 151bp and 133bp band. T/T, C/T and C/C genotypes have “151bp and 133bp bands”, “151bp, 133bp” and 284bp bands”, and “284bp bands” respectively. On the other hand, the A allele of PD-L1(rs2890658) allowed the digestion of the 553bp amplicon into two products of 456bp and 97bp bands. The C allele was characterized by 326bp, 130bp and 97bp. A/A, A/C and C/C genotypes having “456bp and 97bp band”, “456bp, 326bp, 130bp, and 97bp bands” and “326bp, 130bp, and 97bp,” respectively.

Statistical analysis

All the statistical data was performed by using the Excel 2003 (Microsoft Corporation, Redmond, USA) and JMP 16 (SAS Institute, Cary, USA). To determine the Hardy-Weinberg equilibrium (HWE) among the controls, we used a chi-square test. The investigation into the correlation between PD-1 and PD-L1 polymorphism and urothelial cancer was conducted using a multiple logistic regression analysis. We considered a p-value of less than 0.05 to be statistically significant. The multiple logistic regression analysis is one of the statistical methods that can explain and predict the probability of a binary outcome (objective variable) occurring from several factors (explanatory variables).

## Results

In this study, both PD-1(rs2227981) and PD-L1(rs2890658) polymorphisms were investigated using 211 controls and 256 cases of urothelial cancer. There was no difference in the mean age between the cases and controls. However, the mean age of males in the cases was significantly higher than males of controls. On the other hand, smokers were predominant in the case group, and females were predominant in the controls (Table 1). Controls for PD-1 and PD-L1 genes were on the HWB equilibrium (P=0.31 and 0.69, respectively).

**Table 1:** The characteristics of cases and controls We separately examined the cases and controls groups of samples and, variables were age and smoking status of the participants. We evaluated mean age by t-test, and smoking status by chi-square test. *:P<0.05, **:P<0.01

		Cases		Controls	
		Male	Female	Male	Female
Total number (n)		207	49	105	106**
Age(mean±SD)		69.5±10.8	69.0±11.7	67.1±12.4*	69.2±11.5
Smoking Status	Non-smoker	35	39	16	87**
	Smoker	172	10	89	19*

The distribution of the genotypes of the PD-1(rs2227981) and PD-L1(rs2890658) polymorphisms are indicated in Table 2. The C/T genotype of PD-1(rs2227981) was predominant in cases comparing controls (adjusted OR:1.55, 95%CI:1.02-2.35). However, no significant differences were found between cases and controls concerning the genotypes of PD-L1(rs2890658).

**Table 2:** The relationship between urothelial cancer and PD-1(rs2227981) and PD-L1(rs2890658) polymorphisms Adjusted by age, gender, and smoking status *:P<0.05, OR - Odd Ratio

		Case(n=256)	Control(n=211)	Crude OR(95%CI)	Adjusted OR(95%CI)
PD1	C/C	134	130	ref	ref
	C/T	104	68	1.48(1.01-2.19)*	1.55(1.02-2.35)*
	T/T	18	13	1.34(0.63-2.85)	1.15(0.52-2.56)
	C/T+T/T	122	81	1.46(1.01-2.12)*	1.48(1.00-2.19)
PDL1	A/A	218	187	ref	ref
	A/C	36	22	1.41(0.80-2.48)	1.42(0.78-2.60
	C/C	2	1	1.72(0.16-19.17)	1.21(0.11-13.81)
	A/C+C/C	38	23	1.42(0.82-2.48)	1.42(0.79-2.54)

We also evaluated the interaction of PD-1 and PD-L1 polymorphisms (Table 3). We stratified the PD-L1(rs2890658) genotype into A/A and A/C+C/C genotype groups because there were few C/C genotypes. [A/A(PD-L1) and C/T(PD-1)], [A/A (PD-L1) and C/T+ T/T(PD-1)] and [A/A (PD-L1) and C/T+ T/T(PD-1) plus A/C+C/C(PD-L1) and any PD-1 genotype] were significantly higher in the cases. The adjusted ORs were 1.76 (95%CI:1.12-2.74), 1.65 (95%CI:1.08-2.51), and 1.68 (95% CI:1.14-2.48), respectively.

**Table 3:** The relationship between urothelial cell carcinoma and combining PD-1(rs2227981) and PD-L1(rs2890658) polymorphisms We classified the PD-L1(rs2890658) genotype to A/A and A/C+C/C genotype to analyze the relation between each genotype and urothelial cancer. [A/A(PD-L1) and C/T(PD-1)], [A/A (PD-L1) and C/T+T/T(PD-1)] and [A/A (PD-L1) and C/T+ T/T(PD-1) plus A/C+C/C(PD-L1) and any PD-1 genotype] were significantly higher in the case group. **:P<0.01, *:P<0.05, Adjusted by age and smoking status.

PDL1	PD1	Case (n=256)	Control (n=211)	Crude OR (95%CI)	Adjusted OR (95%CI)	Case (n=256)	Control (n=211)	Crude OR (95%CI)	Adjusted OR (95%CI)	Case (n=256)	Control (n=211)	Crude OR (95%CI)	Adjusted OR (95%CI)
A/A	C/C	108	114	ref	ref	108	114	ref	ref	108	114	ref	ref
	C/T	93	61	1.61(1.06-2.44)*	1.76(1.12-2.74)*	110	74	1.57(1.06-2.33)*	1.65(1.08-2.51)*	148	97	1.61(1.12-2.33)*	1.68(1.14-2.48)**
	T/T	17	13	1.38(0.64-1.38)	1.21(0.54-2.73)								
A/C+C/C	C/C	26	16	1.72(0.87-1.72)	1.90(0.92-3.91)	26	16	1.72(0.87-1.72)	1.90(0.92-3.90)				
	C/T	11	7	1.66(0.62-4.44)	1.40(0.50-3.94)	12	7	1.81(0.69-4.77)	1.52(0.55-4.18)				
	T/T	1	0	―	―								

We also analyzed the distribution of PD-1(rs2227981) and PD-L1(rs2890658) polymorphisms stratified by gender and smoking status (Tables 4, 5). Only the male group indicated [A/A (PD-L1) and C/T(PD-1)] and [A/A (PD-L1) and C/T+ T/T(PD-1) plus A/C+C/C(PD-L1) and any PD-1genotype] were also significantly more in the case group, which was similar with the results indicated in Table 3.

**Table 4:** The relationship between urothelial cell carcinoma and combining PD-1(rs2227981) and PD-L1(rs2890658) polymorphisms stratified by gender and smoking status (Part 1) We stratified the male and female group, smoker, and non-smoker group separately. Only the male group indicated [A/A (PD-L1) and C/T(PD-1)] genotype was significant more in case group. **:P<0.01, *:P<0.05. #:Adjusted by age and smoking status ‡:Adjusted by age and gender.

	PDL1	PD1	Case(n=207)	Control(n=105)	Crude OR(95%CI)	Adjusted OR(95%CI)#
Males	A/A	C/C	88	59	ref	ref
		C/T	73	26	1.88(1.08-3.28)*	1.83(1.05-3.21)*
		T/T	15	9	1.12(0.46-2.72)	1.18(0.48-2.91)
	A/C+C/C	C/C	21	6	2.35(0.89-6.16)	2.41(0.91-6.39)
		C/T	9	5	1.21(0.39-3.78)	1.17(0.37-3.70)
		T/T	1	0	―	―
			Case(n=49)	Control(n=106)	Crude OR(95%CI)	Adjusted OR(95%CI)＃
Females	A/A	C/C	20	55	ref	ref
		C/T	20	35	1.57(0.74-3.33)	1.55(0.73-3.30)
		T/T	2	4	1.38(0.23-8.10)	1.35(0.22-8.11)
	A/C+C/C	C/C	5	10	1.38(0.42-4.52)	1.39(0.42-4.58)
		C/T	2	2	2.75(0.36-20.85)	2.89(0.37-22.39)
		T/T	0	0	―	―
			Case(n=74)	Control(n=103)	Crude OR(95%CI)	Adjusted OR(95%CI)‡
Non-smokers	A/A	C/C	29	56	ref	ref
		C/T	29	30	1.87(0.95-3.68)	1.99(0.96-4.13)
		T/T	4	6	1.29(0.34-4.93)	0.91(0.21-4.00)
	A/C+C/C	C/C	10	9	2.15(0.78-5.87)	2.18(0.75-6.38)
		C/T	2	2	1.93(0.26-14.42)	3.00(0.39-23.25)
		T/T	0	0	―	―
			Case(n=182)	Control(n=108)	Crude OR(95%CI)	Adjusted OR(95%CI)‡
smokers	A/A	C/C	79	58	ref	ref
		C/T	64	31	1.52(0.88-2.62)	1.62(0.92-2.85)
		T/T	13	7	1.36(0.51-3.63)	1.36(0.50-3.69)
	A/C+C/C	C/C	16	7	1.68(0.65-4.34)	1.73(0.65-4.60)
		C/T	9	5	1.32(0.42-4.15)	1.13(0.36-3.57)
		T/T	1	0	―	―

**Table 5:** The relationship between urothelial cell carcinoma and combining PD-1(rs2227981) and PD-L1(rs2890658) polymorphisms stratified by gender and smoking status (Part 2) We stratified the male and female group, smoker, and non-smoker group separately. [A/A (PD-L1) and C/T+ T/T(PD-1) plus A/C+C/C(PD-L1) and any PD-1 genotype] was also significant more in the case group among male. **:P<0.01, *:P<0.05. #:Adjusted by age and smoking status ‡:Adjusted by age and gender.

	PDL1	PD1	Case (n=207)	Control (n=105)	Crude OR (95%CI)	Adjusted OR (95%CI)#	Case (n=207)	Control (n=105)	Crude OR (95%CI)	Adjusted OR (95%CI)#
Males	A/A	C/C	88	59	ref	ref	88	59	ref	ref
		C/T	88	35	1.69(1.01-2.81)*	1.67(1.00-2.79)	119	46	1.73(1.08-2.79)*	1.73(1.07-2.78)*
		T/T								
	A/C+C/C	C/C	21	6	2.35(0.89-6.16)	2.42(0.91-6.42)				
		C/T	10	5	1.34(0.44-4.12)	1.31(0.42-4.06)				
		T/T								
			Case (n=49)	Control (n=106)	Crude OR (95%CI)	Adjusted OR (95%CI)#	Case (n=49)	Control (n=106)	Crude OR (95%CI)	Adjusted OR (95%CI)#
Females	A/A	C/C	20	55	ref	ref	20	55	ref	ref
		C/T	22	39	1.55(0.75-3.22)	1.53(0.73-3.20)	29	51	1.56(0.79-3.10)	1.56(0.78-3.09)
		T/T								
	A/C+C/C	C/C	5	10	1.38(0.42-4.52)	1.39(0.42-4.57)				
		C/T	2	2	2.75(0.36-20.85)	2.88(0.37-22.33)				
		T/T								
			Case (n=74)	Control (n=103)	Crude OR(95%CI)	Adjusted OR (95%CI)‡	Case (n=74)	Control (n=103)	Crude OR (95%CI)	Adjusted OR (95%CI)‡
Non- smokers	A/A	C/C	29	56	ref	ref	29	56	ref	ref
		C/T	33	36	1.77(0.92-3.40)	1.78(0.89-3.59)	45	47	1.85(1.01-3.39)*	1.90(0.99-3.64)
		T/T								
	A/C+C/C	C/C	10	9	2.15(0.78-5.87)	2.17(0.75-6.31)				
		C/T	2	2	1.93(0.26-14.42)	2.91(0.38-22.52)				
		T/T								
			Case (n=182)	Control (n=108)	Crude OR (95%CI)	Adjusted OR (95%CI)‡	Case (n=182)	Control (n=108)	Crude OR (95%CI)	Adjusted OR (95%CI)‡
smokers	A/A	C/C	79	58	ref	ref	79	58	ref	ref
		C/T	77	38	1.49(0.89-2.49)	1.57(0.92-2.67)	103	50	1.51(0.94-2.44)	1.56(0.95-2.54)
		T/T								
	A/C+C/C	C/C	16	7	1.68(0.65-4.34)	1.73(0.65-4.60)				
		C/T	10	5	1.47(0.48-4.53)	1.26(0.41-3.92)				
		T/T								

## Discussion

PD-1 protein is an inhibitor of both adaptive and innate immune responses, while PD-L1 protein is expressed by tumor cells as an adaptive immune mechanism to escape anti-tumor response [27]. In addition, the PD-1/PD-L1 function blockage may attenuate apoptosis of CD8-positive T-cells by regulating the PI3K/AKT/mTOR pathway in gastrointestinal tumors [28]. A negative feedback system by inducing active T-cell apoptosis and interleukin-10 (IL-10) was detected in colorectal cancer [29].

We analyzed the relationship between the PD-1(rs2227981) and PD-L1(rs2890658) polymorphisms and urothelial cell carcinoma among a Japanese population. We detected that the C/T genotype of PD-1(rs2227981) was predominant significantly in cases comparing controls (Table 2). However, we did not find any significant differences in the T/T genotype between cases and controls. The reason for this might be that the number of T/T among cases and controls was small. Some researchers have indicated similar tendencies to our results [30-32] that gastric, breast, and cervical cancers had significant in rs2227981 in the PD-1 gene. Conversely, it has been suggested that the C/T genotype of PD-1(rs2227981) could also be a protective factor [33]. A meta-analysis has shown that the T allele of PD-1(rs2227981) decreased the susceptibility to cancer [34]. However, the sample population of this meta-analysis was not Japanese and was not connected to urothelial cell carcinoma. The effects of PD-1(rs2227981) might be different depending on race, type of cancer, and the polymorphism of PD-1.

Besides PD-1(rs2227981), there have been reports concerning other PD-1 polymorphisms, such as rs36084323, rs7421861, rs11568821 and rs2227982. One meta-analysis has indicated that PD-1(rs36084323) polymorphism could be a risk factor for several types of cancer [35].

 On the other hand, the PD-L1 function is a major cause of cancer immunity evasion [20,36] as binding a PD-1 receptor to a PD-L1 receptor can inhibit T-cell proliferation and apoptosis [37]. As a result, this binding can enable carcinogenesis. Some studies have shown that elevated PD-L1 expression of urothelial tissues has been associated with lower survival rates of urothelial cell carcinoma patients [6,38]. Some studies have also indicated that PD-L1(rs10815225, rs4143815, and rs2890658) polymorphism may be related to the carcinogenesis of human cancer, including gastric cancer and lung cancer [39-42]. Additionally, one meta-analysis has also indicated a significant relation between PD-L1(rs4143815) polymorphism and human cancers. However, in our study, we did not notice a significantly different PD-L1(rs2890658) distribution between the case and control groups (Table 2). Similarly, PD-L1 polymorphism(rs4143815) was not associated with breast cancer in the Iranian population [31]. Since ours was the first study of PD-L1(rs2890658) polymorphism using urothelial cell carcinoma cases in the Japanese population, however, we could not compare the results to other Japanese studies. Further, as there have not been many studies conducted concerning PD-L1 polymorphisms, and how the effects of polymorphisms could vary depending on race and type of cancer, additional evaluations will be needed to prove the relation between PD-L1 polymorphisms and human cancer.

In our study, we analyzed the interactions between PD-1(rs2227981) and PD-L1(rs2890658) polymorphisms (Table 3). PD-1/PD-L1 interaction can deregulate T-cell responses and serve as an effective pathway allowing cancer cells to escape immunity surveillance [43,44]. Moreover, since the SNP of PD-L1(rs4143815) is close to or within the transcriptional factor binding sites, polymorphism of PD-L1 may affect the processing and binding affinity [45]. Our results revealed that [A/A (PD-L1) and C/T(PD-1)], [A/A (PD-L1) and C/T+T/T(PD-1)] and [A/A (PD-L1) and C/T+ T/T(PD-1) plus A/C+C/C(PD-L1) and any PD-1genotype] were predominant among the cases. However, OR was not different so much. Since the A/C or C/C genotype of PD-L1(rs2890658) has been reported as a risk factor for the carcinogenesis of human cancer [41,42,46,47], the combination of [A/C+C/C of PD-L1 and C/T or T/T of PD-1] should significantly increase in the cases. However, this study did not indicate any such difference. The reason for this discrepancy was not clear, thus additional research will be needed to evaluate the interaction between PD-1 and PD-L1 polymorphisms. This was the first study concerning the interaction between PD-1 and PD-L1 concerning carcinogenesis.

We also evaluated the interaction between PD-1(rs2227981) and PD-L1(rs2890658) polymorphisms according to gender and smoking status. We obtained the same result as indicated in Table 3 only among the males. Findings concerning the interaction between gene polymorphisms and gender were contradictory. It has been reported that TAP1 polymorphism increases susceptibility in both genders to colon cancers [48]. However, Holipah et al. have reported that the mutant type (G/G) of PER1(rs3027188) is protective against colon cancer in females [49]. Dresler et al. have also reported that the polymorphisms of cytochrome P450 1A1 (CYP1A1) (exon 7) were a risk factor for lung cancer, especially in females [50]. They pointed out that the different effects of polymorphisms by gender were derived from the influence of female sex steroid hormones, such as estrogen. Some studies have further suggested that the estrogen receptor ERα and Erβ expressions were different depending on the kind of cancer [51,52].

On the other hand, although smoking has been regarded as the predominant risk factor for the development of urothelial cell cancer [53,54], our study did not reveal any significant interaction with smoking status. Tobacco smoke contains many carcinogenic compounds that can cause DNA damage, mutations, changes in DNA methylation, and polymorphism in the genome and sequences connected to various types of cancer [55-57]. Hect et al. have carried out research on genomic and bioinformatic approaches regarding the association between cancer and smoking, arguing that most genes had been affected by smoking, while one gene variant, such as polymorphism, did not show any health risk, but still had the potential for tobacco-specific nitrosamine production and cancer development [55].

As a result, although smoking is undoubtedly carcinogenic, the effect on polymorphisms may be minimal. For example, Kuroda et al. [58] have found that the Pro allele of the p53 codon 72 polymorphism increases in urothelial cancer cases in lighter smokers, while Khoury et al. [59] and Wang et al. [60] found that genetic differences in cancer risk may be smaller with high carcinogen loads, which is why our findings did not indicate any relationship between smoking and polymorphisms.

There are, however, several limitations to this research. First, we did not have any information about other potential confounding factors as causes of cancer, such as alcohol consumption, the smoking period, the number of cigarettes, and family history of diseases. Neither did our study assess the tumor stage, location, pathological type, or prognosis. Furthermore, since we were required to adopt hospital controls, we cannot rule out the possibility that our controls may contain some kind of bias. Despite these limitations, we believe that this study was the first related to PD-1(rs2227981) and PD-L1(rs2890658) polymorphisms and urothelial cell carcinoma conducted among the Japanese population and therefore may be beneficial in understanding the relationship and interactions between PD-1 and PD-L1 polymorphisms with urothelial cell carcinoma.

## Conclusions

This study evaluated the distribution of PD-1(rs2227981) and PD-L1(rs2890658) polymorphisms between urothelial cell carcinoma cases and controls, and the interaction with PD-1(rs2227981) and PD-L1(rs2890658) polymorphisms. We found that the C/T genotype and [A/A (PD-L1) and C/T(PD-1)] could be a risk factor for urothelial cell carcinoma. However, we could not detect the genetic interaction of PD-1 and PD-L1 polymorphisms. Therefore, although this study was the first concerning the relationship between Japanese urothelial cell cancer and PD-1(rs2227981) and and PD-L1(rs2890658) polymorphisms, additional research will be needed to further evaluate the relationship between human cancer and PD-1 and PD-L1 polymorphisms.
